# Field Evaluation of a Point-of-Care Circulating Cathodic Antigen (POC-CCA) Test for the Detection of *Schistosoma japonicum* in Indonesia

**DOI:** 10.4314/ejhs.v36i1.7

**Published:** 2026-01

**Authors:** Gunawan, Joko Prastowo, Wisnu Nurcahyo, Pandji Wibawa Dhewantara, Anis Nur Widayati, Hayani Anastasia, Samarang, Made Agus Nurjana, Muhammad Yusuf, Siti Soidah, Fauzian Giansyah Rohmatulloh, Sunardi, Yuyun Srikandi

**Affiliations:** 1 Research Centre of Public Health and Nutrition, National Research and Innovation Agency Republic of Indonesia, Indonesia; 2 Doctoral Program Study of Veterinary Science, Faculty of Veterinary, Gadjah Mada University, Indonesia; 3 Department of Veterinary Parasitology, Faculty of Veterinary, Gadjah Mada University, Indonesia; 4 Center for Molecular Biotechnology and Bioinformatics Research, Padjajaran University, Indonesia; 5 Directorate General of Disease Prevention and Control, Ministry of Health, Indonesia; 6 Donggala Public Health Laboratory, Ministry of Health, Donggala, Indonesia

**Keywords:** Schistosomiasis, Schistosoma japonicum, POC-CCA, diagnostic accuracy, Indonesia

## Abstract

**Background:**

Schistosomiasis caused by Schistosoma japonicum remains a public health concern in low-endemic areas of Indonesia Sensitive and field-applicable diagnostic tools are essential for effective surveillance and control This study evaluated the diagnostic performance of a point-of-care circulating cathodic antigen (POC-CCA) urine test for detecting S. japonicum infection.

**Methods:**

A community-based cross-sectional diagnostic evaluation was conducted during August–September 2023 among 226 residents of Wanga (Lore Peore) and Tamadue (Lore Timur) villages, Poso Regency, Central Sulawesi, Indonesia Stool samples were examined using the Kato–Katz (KK) technique, and urine samples were tested using the POC-CCA cassette. Diagnostic accuracy indices were calculated using MedCalc software, with KK serving as the reference standard.

**Results:**

Of the 226 participants, 36 (15.9%) were positive for S. japonicum by KK, whereas only 6 (2.7%) tested positive by POC-CCA. A total of 188 participants were negative by both methods, while 30 were KK-positive but POC-CCA–negative. The sensitivity of the POC-CCA test was 16.67% (95% CI: 6.37–32.81), and specificity was 98.95% (95% CI: 96.25–99.87). The positive predictive value was 75.00% (95% CI: 38.66–93.46), the negative predictive value was 86.24% (95% CI: 84.40–87.89), and Cohen's kappa coefficient was 0.246 (95% CI: 0.004–0.487), indicating fair agreement.

**Conclusions:**

The POC-CCA urine test demonstrated high specificity but very low sensitivity for detecting S. japonicum, particularly in low-intensity infections. Its use as a standalone diagnostic tool in low-endemic settings is not recommended Refinement of the assay or integration with complementary diagnostic methods is necessary before field application.

## Introduction

Schistosomiasis is a chronic parasitic disease caused by vascular worms of the genus Schistosoma (1). The disease poses a major public health challenge and causes substantial economic losses in many developing countries. Schistosomiasis occurs predominantly in tropical and subtropical regions, particularly among impoverished communities lacking access to clean water and adequate sanitation (1–5). Chronic infection reduces work capacity and, in severe cases, can be fatal. In children, schistosomiasis is associated with growth retardation, anemia, and impaired cognitive development (6).

Schistosomiasis manifests in two main clinical forms: urogenital and intestinal. Urogenital schistosomiasis is caused by Schistosoma haematobium (7), while intestinal schistosomiasis is caused by S. mansoni, S. japonicum, S. mekongi, S. intercalatum, and S. guineensis (4,8). Six Schistosoma species are known to infect humans: S. mansoni, found in Africa, South America, the Caribbean, and the Middle East; S. haematobium, found mainly in Africa and the Middle East, with recent autochthonous transmission reported in Corsica, France; S. intercalatum and S. guineensis, which occur in parts of Central Africa; S. japonicum, endemic to Asia including China, Indonesia, and the Philippines; and S. mekongi, found in the Mekong Delta region, including Cambodia and the Lao People's Democratic Republic (Laos) (9,10).

Globally, schistosomiasis affects approximately 240 million people, with an estimated 700 million individuals living in endemic areas across 78 countries. According to the World Health Organization (WHO), at least 229 million people required preventive chemotherapy in 2018 (8). Schistosomiasis has a long history in Asia. Although the disease was first formally described in the early twentieth century, historical evidence suggests that it was endemic in Japan at least 400 years earlier and in the People's Republic of China for more than 2,200 years. In China, schistosomiasis was initially identified in ancient mummies (9). Japan remains the only Asian country to have successfully eliminated schistosomiasis, while Thailand is awaiting WHO confirmation of transmission interruption (11).

In Indonesia, schistosomiasis is caused exclusively by S. japonicum. In endemic communities, the disease is commonly referred to as “snail fever.” In addition to humans, S. japonicum infects a wide range of domestic and wild mammals. Thirteen mammalian species have been reported as hosts, including cattle (Bos sundaicus), buffalo (Bubalus bubalis), horses (Equus caballus), dogs (Canis familiaris), pigs (Sus spp.), ferrets (Viverra tangalunga), deer (Cervus timorensis), and several rodent species (Rattus exulans, R. marmosurus, R. norvegicus, and R. palellae). Among these, livestock such as cattle, buffaloes, horses, and dogs are considered important reservoir hosts for S. japonicum (12–17).

Unlike in the People's Republic of China and the Philippines, schistosomiasis in Indonesia is geographically restricted to Central Sulawesi Province, specifically the Napu and Bada Plateaus in Poso Regency and the Lindu Plateau in Sigi Regency (16,17). Although the prevalence of S. japonicum infection has fluctuated over recent decades, it has generally remained higher in the Napu Plateau and has shown an overall increasing trend. Control efforts in Central Sulawesi have substantially reduced schistosomiasis prevalence since 2005; however, fluctuations persist in the Napu, Lindu, and Bada Plateaus. Ministry of Health reports indicate that human prevalence in Poso Regency remained relatively low between 2018 and 2021 (0.10%–0.36%) but increased to 1.81% in 2022 (10).

Several diagnostic methods are available for the detection of schistosomiasis, including parasitological, molecular, and immunological approaches. Parasitological diagnosis involves direct microscopic examination of stool samples for Schistosoma eggs. The World Health Organization recommends the Kato–Katz (KK) technique as the standard parasitological method for schistosomiasis diagnosis. The KK method is widely used in control programs because it is inexpensive and relatively simple to perform; however, its sensitivity is limited in areas of low prevalence or low-intensity infection (18, 19). Alternative parasitological techniques include formalin–ethyl acetate sedimentation (FEA-SD) (20), the Danish Bilharzia Laboratory (DBL) technique, and the miracidium hatching technique (MHT), which also assesses egg viability (21,22). Molecular diagnostic methods detect parasite DNA in clinical samples, commonly stool but also urine, blood, and saliva, and include loop-mediated isothermal amplification (LAMP), conventional polymerase chain reaction (cPCR), real-time PCR (qPCR), and digital droplet PCR (ddPCR) (23).

Detection of schistosomal antigens derived from schistosomula, adult worms, or eggs in blood, urine, or sputum has emerged as a reliable diagnostic approach. Commonly targeted antigens include adult worm antigen (AWA), soluble egg antigen (SEA), and circulating antigens. The two most extensively studied circulating antigens are circulating cathodic antigen (CCA) and circulating anodic antigen (CAA). CCA- and CAA-based assays can estimate active worm burden and are useful for monitoring treatment response. Both antigens become detectable in blood approximately three weeks after infection and are subsequently excreted in urine. They can be detected using enzyme-linked immunosorbent assays (ELISA) in serum and urine samples, with higher sensitivity typically achieved for CAA in serum and CCA in urine. Consequently, these assays are suitable for use in endemic settings as well as in areas of low endemicity. The detection of CCA in urine has been developed into a rapid lateral flow cassette test for the diagnosis of intestinal schistosomiasis caused by S. mansoni (23).

This study aimed to evaluate the performance of the point-of-care circulating cathodic antigen (POC-CCA) urine test for detecting S. japonicum infection in areas of low schistosomiasis endemicity in Indonesia.

## Materials and Methods

**Study design and setting**: A community-based cross-sectional diagnostic evaluation was conducted between August and September 2023 in Wanga (Lore Peore) and Tamadue (Lore Timur) villages, Poso Regency, Central Sulawesi, Indonesia. A census-based recruitment approach was applied, whereby all eligible residents were invited to participate.

**Sample size**: The minimum sample size was calculated using a single-proportion formula, assuming a prevalence of 4.48%, a 95% confidence level, and a precision of 3%. After adjusting for potential non-response, the target sample size was 229. A total of 226 participants provided complete and valid samples and were included in the analysis.

**Sample collection**: Participants were requested to provide three stool samples on consecutive days and one urine sample. Sociodemographic information was collected using a structured questionnaire administered by trained interviewers.

**POC-CCA urine test**: Urine samples were tested using the POC-CCA cassette (Rapid Medical Diagnostics, Pretoria, South Africa) according to the manufacturer's instructions. Results were read after 20 minutes and classified as negative, trace, or positive based on the presence of test and control lines.

**Parasitological examination**: Stool samples were examined using the Kato–Katz technique. Three thick smears (41.7 mg each) were prepared from each stool sample, resulting in nine slides per participant. Slides were examined microscopically by experienced laboratory personnel within 24 hours of preparation.

**Data analysis**: Diagnostic performance indices—including sensitivity, specificity, positive predictive value, and negative predictive value—were calculated using MedCalc software. Agreement between diagnostic methods was assessed using Cohen's kappa coefficient.

**Ethical considerations**: Ethical approval was obtained from the Research Ethics Committee of the National Research and Innovation Agency (Approval No. 026/KE.03/SK/04/2023). Written informed consent was obtained from all participants prior to enrollment.

## Results

**Characteristics of the study population:** A total of 226 residents participated in the study. Sociodemographic characteristics of the respondents are presented in Table 1.

**Table 1:** Characteristics of respondents

Characteristics	N	%
Age		
< 15 years	56	24.8
15-30 years	27	11.9
31-50 years	90	39.8
> 50 years	53	23.5
Gender		
Male	121	53,5
Female	105	46,5
Education		
No/not yet in school	9	4,0
Does not end	41	18,1
Elementary school equivalent	53	23,5
Junior high school equivalent	58	25,7
High school equivalent	53	23,4
Academy/PT	12	5,3
Work		
PNS/TNI/Polri/BUMN	6	2,7
Wiraswasta	5	2,2
Farmer	94	41,8
Does not work	18	8,0
Student	54	24,0
Household affairs	39	17,3
Other	9	4,0

**Parasitological findings**: Using the Kato–Katz method, 36 participants were positive for S. japonicum. According to WHO criteria, 35 infections were classified as light intensity and one as heavy intensity. The distribution of infection intensity is shown in Table 2.

**Table 2:** *S. japonicum* infection intensity

		Infection Intensity (KK)		
Group	n	Light (EPG: 1-99)	Moderate (EPG: 100-399)	Heavy (EPG:>400)
KK(+)	36	35	0	1
KK(-)	190	0	0	0

EPG: Egg per gram of faeces, KK: Kato-Katz

**Figure 1:**
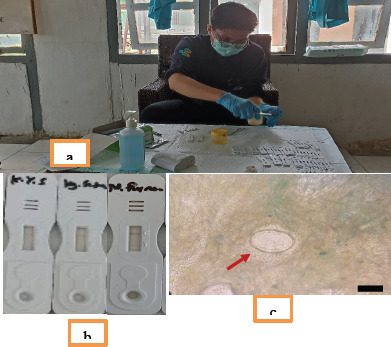
Sample collection and processing (a), representative positive and negative results of the POC-CCA test (b), typical egg of Schistosoma japonicum observed by Kato–Katz thick smear (c). Scale bar = 50 µm

**Diagnostic performance of the POC-CCA test**: Of the 226 samples analyzed, 6 were positive by POC-CCA, while 36 were positive by Kato–Katz. A total of 188 participants were negative by both methods. Thirty participants were KK-positive but POC-CCA–negative, and two were POC-CCA–positive but KK-negative (Figure 1).

The sensitivity of the POC-CCA test was 16.67% (95% CI: 6.37–32.81), and specificity was 98.95% (95% CI: 96.25–99.87). The positive predictive value was 75.00% (95% CI: 38.66–93.46), the negative predictive value was 86.24% (95% CI: 84.40–87.89), and overall diagnostic accuracy was 85.84% (95% CI: 80.60–90.11). Cohen's kappa coefficient was 0.246, indicating fair agreement (Table 3).

**Table 3:** The performance evaluation results of the POC CCA test

POC-CCA	Reference test		Sensitivity, % (95% CI)	Specificity, % (95%CI)	PPV, % (95%CI)	NPV, % (95%CI)	Accuracy, % (95% CI)	Kappa index (95%CI)
	+	-						
	KK							
+ -	6 30	2 188	16.67 (6.37-32.81)	98.95 (96.25-99.87)	75 (38.66-93.46)	86.24 (84.40-87.89)	85.84 (80.60-90.11)	0.246 (0.004-0.487)

Cl Confibence Interval, KK Kato–Katz, POC-CCA Point-of-care circulating cathodic antigen, PPV positive predictive value, NPV negative predictive value

## Discussion

Since 2008, a commercially available lateral flow cassette urine test for the detection of circulating cathodic antigen (CCA) released by adult schistosomes has been widely used. Several studies have reported that the point-of-care CCA (POC-CCA) test is considerably more sensitive than the Kato–Katz (KK) stool examination for detecting S. mansoni infection, particularly in low-endemic settings. The POC-CCA test has been extensively validated for the rapid diagnosis of schistosomiasis, especially among individuals infected with S. mansoni. Satisfactory diagnostic performance has been reported in areas endemic for S. mansoni where the prevalence determined by the KK method is below 50%. In such settings, prevalence estimates obtained using POC-CCA have been reported to be 1.5–6 times higher than those derived from the KK technique (24,25).

The schistosomiasis control strategy in Indonesia was developed by the Ministry of Health—through the Sub-Directorate of Filariasis and Schistosomiasis, the Directorate General of Disease Prevention and Control (P2P), and the Health Research and Development Agency—in collaboration with the Central Sulawesi Provincial Health Office for the period 2011–2016. The strategy aimed to achieve at least 85% coverage of mass drug administration in high-endemic areas, implement active selective treatment in medium-endemic areas, and apply passive selective treatment in low-endemic areas. Preventive chemotherapy was complemented by interventions targeting livestock treatment, snail control, health education, improvements in water supply and sanitation, and strengthened monitoring, evaluation, and health facility development in endemic areas. Despite substantial progress in reducing schistosomiasis prevalence, control and prevention efforts have been constrained by limited intersectoral coordination, as well as insufficient financial and human resources (16,26–29).

In Indonesia, the diagnosis of schistosomiasis still relies primarily on microscopic examination of stool samples. The KK technique is regarded as the reference or gold standard for diagnosing human schistosomiasis because of its simplicity, low cost, and high specificity when performed by trained laboratory personnel (16,27,30). However, its application is limited by very low sensitivity in settings with low transmission intensity or low endemicity (31). Consequently, there is a clear need to evaluate alternative and complementary diagnostic methods for schistosomiasis surveillance in Indonesia.

In this study, we evaluated the diagnostic performance of a point-of-care circulating cathodic antigen (POC-CCA) urine test for the detection of S. japonicum. Among the 226 samples analyzed, microscopic examination identified 36 individuals as positive for S. japonicum, whereas only six individuals tested positive using the POC-CCA assay. A total of 188 samples were negative by both diagnostic methods. In contrast, six samples were positive by POC-CCA alone, while 30 samples were positive by microscopy but negative by POC-CCA. The sensitivity of the POC-CCA test was 16.67% (95% CI: 6.37–32.81), and its specificity was 98.95% (95% CI: 96.25–99.87). The positive predictive value was 75.00% (95% CI: 38.66–93.46), and the negative predictive value was 86.24% (95% CI: 84.40–87.89). The Cohen's kappa coefficient was 0.246 (95% CI: 0.0043–0.4877), indicating low agreement between the POC-CCA assay and microscopic examination. These findings highlight the limited sensitivity of the POC-CCA test and underscore the need for improved or supplementary diagnostic approaches.

Our findings are consistent with those reported by Cai et al. (2021), who demonstrated that the POC-CCA test has limited sensitivity for detecting S. japonicum infection, particularly among individuals with low-intensity infections (32). Similarly, Hoermann et al. (2022) reported that although POC-CCA may be useful as a point-of-care tool, its diagnostic performance is strongly influenced by infection intensity and the level of endemicity. Both studies emphasized the limitations of antigen-based assays in low-endemic settings, which aligns with our observation that POC-CCA failed to detect a substantial proportion of KK-positive cases. Collectively, these findings support the conclusion that the POC-CCA test, in its current form, is inadequate as a stand-alone diagnostic tool for S. japonicum in Indonesia. Combined diagnostic approaches, incorporating parasitological and molecular methods, may provide more reliable data for surveillance and control (33).

Recent advances in antigen detection offer promising alternatives. Colley et al. (2023) reported that detection of circulating anodic antigen (CAA) in serum provides superior sensitivity compared with CCA detection in urine, particularly in low-endemic settings. Although CCA-based urine assays such as POC-CCA remain attractive for field use due to their operational feasibility, their current performance for detecting S. japonicum remains suboptimal (34). Integrating CCA-based testing with more sensitive diagnostic tools, including serum CAA detection or molecular assays such as PCR or qPCR, may substantially enhance surveillance accuracy in endemic areas of Indonesia.

The strengths of this study include its community-based design and the use of multiple stool samples with replicated Kato–Katz smears, which increased overall diagnostic accuracy. Nevertheless, several limitations should be acknowledged. Molecular diagnostic methods, such as PCR, were not included as reference tests due to logistical constraints, which may have resulted in an underestimation of true infection prevalence. In addition, the poor sensitivity of POC-CCA observed in this study may reflect species-specific differences in antigen expression or excretion, warranting further investigation.

In conclusion, the POC-CCA urine test demonstrated high specificity but poor sensitivity for detecting S. japonicum infection in low-endemic areas of Central Sulawesi. Although it cannot currently replace parasitological or molecular diagnostic methods, further refinement of the assay or its integration with complementary diagnostic approaches is essential to achieve reliable field surveillance and support schistosomiasis elimination efforts in Indonesia.
